# Diverse functions of miR-125 family in different cell contexts

**DOI:** 10.1186/1756-8722-6-6

**Published:** 2013-01-15

**Authors:** Yu-Meng Sun, Kang-Yu Lin, Yue-Qin Chen

**Affiliations:** 1 Key Laboratory of Gene Engineering of the Ministry of Education, State Key Laboratory for Biocontrol, School of Life Science, Biotechnology Research Center, Sun Yat-sen University, Guangzhou 510275, People’s Republic of China; 2 Northwest A & F University, Yangling, Shanxi 712100, China

**Keywords:** miR-125 family, Biomarker, Immune response, Therapeutic target, Tumor-suppressor, Tumor-promoter

## Abstract

MicroRNAs (miRNAs) are emerging as a novel class of non-coding RNA molecules that regulate gene expression at a post-transcriptional level. More than 1000 miRNAs have been identified in human cells to date, and they are reported to play important roles in normal cell homeostasis, cell metastasis and disease pathogensis and progression. MiR-125, which is a highly conserved miRNA throughout diverse species from nematode to humans, consists of three homologs hsa-miR-125a, hsa-miR-125b-1 and hsa-miR-125-2. Members of this family have been validated to be down-regulated, exhibiting its disease-suppressing properties in many different types of diseases, while they also have disease-promoting functions in certain contexts. MiR-125 targets a number of genes such as transcription factors, matrix-metalloprotease, members of Bcl-2 family and others, aberrance of which may lead to abnormal proliferation, metastasis and invasion of cells, even carcinomas. Furthermore, miR-125 plays a crucial role in immunological host defense, especially in response to bacterial or viral infections. In this review, we summarize the implication of miR-125 family in disease suppression and promotion, focusing on carcinoma and host immune responses. We also discussed the potential of this miRNA family as promising biomarkers and therapeutic targets for different diseases in future.

## Introduction

MicroRNAs (miRNAs) are a family of ~22 nucleotide (nt) small noncoding RNAs that act as crucial posttranscriptional regulators of gene expression through translational repression or transcript cleavage [1]. Since miRNAs were first described as endogenous mediators in *C. elegans* in 1993 [2], they have been identified to be involved in the regulation of numerous cellular processes, including cell differentiation, proliferation, apoptosis and metabolic homeostasis [3]. In the past decade, more and more studies have demonstrated that aberrant expression of miRNAs is tightly related to the pathogenesis of diseases, including almost all types of human cancers [4]. A growing body of studies elucidate that miRNAs can function as either tumor suppressors by down-regulating oncogenic targets, or tumor promoters through negatively regulating tumor-suppressive target mRNAs [5]. Because of aberrant expression of miRNAs in almost all types of cancers and other diseases, they can possibly be used as biomarkers for early diagnosis of cancers and other diseases, depending on their abundance. In addition, miRNAs and their target genes supply to a general or personalized therapy with pharmaceutical targets, showing their remarkable value in clinical therapy.

Among the most important miRNA families, miR-125 family has been reported to be implicated in a variety of carcinomas and other diseases as either repressors or promoters. MiR-125 family is composed of three homologs hsa-miR-125a, hsa-miR-125b-1 and hsa-miR-125-2. MiR-125a has been found to be located at 19q13, while miR-125b is verified to be transcribed from two loci located on chromosomes 11q23(hsa-miR-125b-1) and 21q21(hsa-miR-125b-2) [6]. Furthermore, miR-125b-1 is implicated in some chromosomal translocations like t(11;14)(q24;q32) and t(2;11)(p21;q23) which leads to B-cell acute lymphoid leukemia (B-ALL) or myelodysplasia and acute myeloid leukemia (AML), respectively [7-10]. Members of the family play crucial roles in many different cellular processes like cell differentiation, proliferation and apoptosis by targeting many different transcription factors [11], matrix-metalloprotease [12,13], growth factors [14] and so on. The theme of the present paper, however, is to summarize the function of miR-125 family in different contexts, particularly in disease condition. We mainly focus on following four aspects: (1) the regulation of miR-125 at post-transcription level, (2) the function of miR-125 as tumor-suppressive or tumor-promoting properties, (3) potential acting of miR-125 as biomarker, and its possibility to be used as a therapeutic target in disease therapy, and (4) the immune-modulating functions of miR-125 and the involvement of this miRNA in immune responses to pathogen infections.

### Involvement of the miR-125 family in solid tumors

A great amount of studies have investigated the relationships between miRNAs and malignancies, and the results show that miRNA deregulation is involved in all types of cancer. The different members of miR-125 family have been reported controversial properties in different types of cancer; they may contribute to the initiation and progression of cancers by acting as either tumor suppressors or oncogenes [15-18].

MiR-125 has been shown its tumor-suppressor functions in several cancers including ovarian cancer [16,19], bladder cancer [20], breast cancer [21-23], hepatocellular carcinoma [12,24,25], melanoma [26], cutaneous squamous cell carcinoma [13] and osteosarcoma [27]. Wang et al. reported that miR-125a, activated by EGFR, functions as a metastatic suppressor in lung cancer cells, inhibiting tumor formation and tube formation [28]. In breast cancer, miR-125a and miR-125b were reported down-regulated in biopsy specimens and as tumor suppressors [23,29,30] by mediating the ERBB2 and ERBB3 pathway [22] or by targeting the ETS1 gene [31]. Furthermore, Hajabi et al. found that miR-125b can reduce the expression of MUC1 oncoprotein, silencing of which in breast cancer cells with siRNA promotes DNA damage-induced apoptosis [32]. Ectopic expression of both miR-125a [12] and miR-125b [25] can inhibit the proliferation and metastasis of hepatocellular carcinoma. Up-regulated miR-125a significantly inhibits the malignant phenotypes by repressing the expression of matrix metalloproteinase 11 (MMP11) and vascular endothelial growth factor A (VEGF-A) both in vitro and in vivo [12], while the direct targets of miR-125b in miRNA-induced inhibition of hepatocellular carcinoma cell proliferation are Mcl-1 and IL6R [25].

In contrast to the tumor-suppressive properties mentioned above, the members of miR-125 family, especially miR-125b, also act as oncogene in several cancers. Jiang and colleagues observed that AN3CA cells, a type II endometrial carcinoma cell line, transfected with miR-125b mimic showed higher tumorogenesis in comparison to other negative controls in nude mice, suggesting that up-regulated miR-125b can promote the proliferation and migration of the disease [18]. In addition, overexpression of miR-125b has been investigated in several cancers such as pancreatic cancer [33], prostate cancer [34,35], oligodendroglial cancer [36] and other cancers. Furthermore, miR-125b has been demonstrated to play a key role in the initiation and development of prostate cancer through evaluating the effect of miR-125b on xenograft tumor growth in transplanted mice with PC-346C-miR-125b cells [35]. Albeit miR-125b has been demonstrated to be tumor suppressor in breast cancer by down-regulating ERBB2 and ERBB3 [22], several studies have showed its property to increase resistance of cancer cells, including breast cancer cells, to anticancer drug [37], resulting in subsequent recurrence and metastasis. Surprisingly, in contrast to tumor-suppressive function in breast cancer mentioned above, a very recent study by Tang et al. has verified that miR-125b also induces metastasis of human breast cancer cells through targeting STARD13 [38]. miR-125a also enhances invasive potential in urothelial carcinomas [39] and is up-regulated in basal cell carcinoma compared with adjacent nonlesional skin [40], while miR-125b suppresses Bmf-dependent apoptosis in human glioblastoma multiforme cells [41].

The controversial properties of the miR-125 family in different solid tumors suggest that miR-125 plays diverse functions in cancer pathogenesis and progression, while the underlying mechanisms on different cell context need further investigation.

### Properties of the miR-125 family in hematological malignancies

Dysregulation of miRNAs has now clearly been linked to hematological malignancies, especially to leukemia [42]. It has been shown that miR-125b is implicated in some subtypes of leukemia induced by chromosomal translocation. The chromosomal translocation t(11;14)(q24;q32) found in patients with B-cell acute lymphoblastic leukemia (ALL)[7,9] leads to an up-regulation of miR-125b. Moreover, overexpression of miR-125b also occurs in patients with myelodysplasia and acute myeloid leukemia (AML) carrying the t(2;11)(p21;q23) translocation [8]. MiR-125b has also been reported to be overexpressed in megakaryoblastic leukemia [43] and APL [44]. Bousquet and colleagues transplanted fetal liver cells which ectopic express miR-125b into nude mice to decipher oncogenic mechanism of miR-125b [15]. In this study they found a higher level of miR-125b increased white blood cell count in mice and half of these mice died of diverse leukemia such as B-cell and T-cell ALL, or a myeloproliferative neoplasm, suggesting miR-125b an important role in early hematopoiesis (the function then was proved by Chaudhuri [45]). Furthermore, this study also demonstrated that miR-125b can shorten the latency of BCR-ABL induced leukemia as a secondary event. Enomoto et al. reported that transduction of miR-125b-1 in bone marrow cells indeed accelerated myeloid tumors induced by a C-terminal mutant of CAAT-enhancer binding protein (C/EBPα-C(m)) [46], suggesting that overexpression of miR-125b collaborates with other genetic alterations in the pathogenesis of myeloid malignancies. Albeit Enomoto didn’t find leukemia transformation in their model, a recent study by Bousquet has highlighted the transformation of miR-125b-induced leukemia in nude mice [11]. Much to our surprise, miR-125b, which has been shown reduced expression in both aggressive and indolent chronic lymphocytic leukemias (CLL) patients, its overexpression can change metabolic pathways including glucose, glutathione, lipid and glycerolipid metabolism in CLL, suggesting that miR-125b plays a vital role for the adaptation of cell metabolism to a transformed state [47]. However, the mechanism underlying differential expression pattern of miR-125b between CLL and other types of hematological malignancies is still unknown. We speculated that the induced expression of miR-125b in CLL leads to metabolic adaptation associated cancer transformation may be caused by very low proliferative index and defects in apoptotic pathways in the disease, while the up-regulated miR-125b leads to acute leukemia may be resulted from hematopoietic stem cell differentiation block by miR-125b. These speculations need to be further validated.

### The functional link of the miR-125 family in autoimmune diseases

The aberrant expression of miR-125 was also found in different diseases other than cancers, such as autoimmune diseases [48]. For instance, miR-125a has been identified down-regulated in systemic lupus erythematosus (SLE), and negatively regulated expression of the inflammatory chemokine RANTES, which is highly expressed in SLE samples and plays a key role in inflammatory progress [49]. MiR-125a was also reported to be significantly up-regulated in macrophases following oxidized low density lipoprotein (oxLDL) [50], inducing the formation of ischemic stroke [51]. Moreover, miR-125b acting as pro-inflammation role can be mediated by metal sulfates to affect the outcome of ischemic stroke [51,52]. Interestingly, Zhang and colleagues have found the overexpression of miR-125b in eosinophilic chronic rhinosinusitis with nasal polyps, enhancing type I IFN expression through suppressing 4E-BP1 protein expression [53]. In addition, dysregulation of miR-125b has been reported to be involved in Alzheimer's disease [54] and Myotonic Dystrophy Type 2 [55]. All together suggest that miR-125 plays important roles in autoimmune diseases. However, thus far, most of the studies on autoimmune diseases mainly focus on the expression pattern of miR-125, the targets and inflammatory pathway in this disease still need to be declared

### MiR-125 involved in immune system development and immunological host defense

It has been uncovered that miRNAs are tightly associated with immune system by controlling the destiny of immune cells [56,57], regulating the expression of target encoding genes, responding to stimulatory cues, and enhancing the response of immune cells to potential antigen as well as playing a key role in fate of immune cells. An example is that, B lymphocyte-induced maturation protein-1 (BLIMP-1) and IFN regulatory protein-4 (IRF4) transcription factors, which are essential for plasma cell differentiation, have been demonstrated to be targets for miR-125b. Gururajan and colleagues have evaluated the effect of overexpression of miR-125b on B cell differentiation in an LPS-responsive B cell line, finding that miR-125b can repress the differentiation of primary B cell, in which they found that miR-125b promotes B lymphocyte diversification in germinal centers by inhibiting premature utilization of essential transcription factors for plasma cell differentiation [56]. In addition, miR-125b is confirmed to protect B cells from apoptosis through contributing to repression of BCL2 which does a developmental stage-specific regulation during B-cell maturation, and deregulation of miR-125b in response to CD40 ligand (CD154) can lead to proliferation in leukemic B cells [57].

Beyond regulated by cytokine like IL-4 [58] and interferon, miR-125b have also been verified to frequently regulate expression of signals involved in immune system. Studies have showed that up-regulated expression of miR-125b may enhance type I IFN expression in airway epithelial cells in eosinophilic chronic rhinosinusitis with nasal polyps [53], while negatively regulating TNF-α expression in neonatal monocytes[59]. Both signals play crucial roles in immunological host defense, aberrant expression or response of which may result in fatal diseases. Furthermore, overexpression of miR-125b in macrophage showed the repression to IFN regulatory factor 4 (IRF4), and elevated responsiveness to IFN-γ, potentiating the functional role of macrophages in inducing immune responses [60].

Another important function of miR-125 is in immune responses to bacterial infection, and even shows to partake in damage accompanied by pathogenic bacteria. Rajaram has illustrated that virulent mycobacterium tuberculosis could take advantage of miR-125b to block TNF biosynthesis. Lipomannan from virulent mycobacterium tuberculosis induces overexpression of miR-125b, which subsequently binds to the 3' UTR region of TNF mRNA and destabilizes the transcript [61]. Apart from immune reaction to bacterial infection, a study shows that miR-125b plays an important role in anti-viral defense [62]. MiR-125a interacted with the viral sequence and markedly suppressed the reporter activity. Subsequently miR-125a was shown to interfere with the viral translation, down-regulating the expression of the surface antigen [62]. Interestingly, miR-125b has been verified to contribute to HIV-1 latency [63], acting as an anti-HIV-1 miRNA [64]. MiR-125b, repressing HIV-1 activity, is higher expressed in resting CD4+ T cells in comparison to activated CD4+ T cells. The miRNA can bind to the 3' ends of HIV-1 messenger RNAs, leading to destabilization of the transcript, terminally inhibits HIV-1 production and prolongs HIV-1 latency in resting CD4+ T cells [63]. In addition, a recent study has found that morphine was shown to down-regulate the expression of anti-HIV miRNAs including miR-125b in cultured human monocytes [64], which suggests that morphine may have potential to activate latent HIV-1 in resting CD4+ T cells and macrophages by inhibiting related miRNAs expression.

In conclusion, miR-125 family plays crucial roles in immune system development and immunological host defense, including regulating differentiation of immune cells, responding to stimulatory cues in many different signal pathways involved in immune system, controlling expression of target genes, products of which partake in immune reactions as well as in immune responses to bacterial infection and to viral infection.

### The regulatory network of miR-125 family in disease pathogenesis

As previous reported, miR-125 family plays an important role in normal cell homeostasis, cell metastasis and different diseases. Current progression also revealed a number of the miR-125 family target genes and their regulation pathways, which could supply the feasibility of using miR-125 as a therapeutic strategy to suppress diseases.

Members of Bcl-2 family and ones else involved in apoptosis are an important group of miR-125 targets. Anti-apoptotic members of Bcl-2 family such as Bcl-w [65], Bcl-2 [66], Mcl-1 [65,67], and Bak-1 [11,44,68,69] acting as the Bcl-2 homologous antagonist, and others involved in apoptosis like P53 [69], TP53INP1 [11,18], TNFAIP3 [70], p38α [71] et al., have all been demonstrated to be direct targets of miR-125 in previous studies. Aberrant expression of miR-125 leads to up-regulation of Bcl-2, Mcl-1 et al. and down-regulation of Bak-1, TP53INP1 et al. and consequently to protect cells from apoptosis, which in turn promotes tumorigenesis. Besides, p38α expression repressed by miR-125b is required for protecting cells from UV-induced apoptosis.

ERBB2, which enhances kinase-mediated activation of downstream signaling pathways such as MAPK, has been verified to be the target for both miR-125a and miR-125b [22]. Experimentally, miR-125-induced down-regulation of ERBB2 and ERBB3 has been uncovered to reduce cell motility and invasiveness of numerous cancers, including breast cancer [22] and endometrial cancer [72].

Among other targets of miR-125 associated with proliferation, metastasis and migration, HuR [73], Rock-1 [74], PDPN [75], STAT3 [27] and STARD13 [38] are five important genes identified, which can induce cell metastasis and migration, and in turn enhance tumorigenesis. Furthermore, CBFβ, a transcriptional factor involved in hematopoiesis, which has been confirmed to be a new direct target of miR-125b, and ABTB1, an anti-proliferative factor targeted by miR-125b, also contribute to block differentiation and proliferation in leukemia, respectively [11,76].

Other targets have been demonstrated including matrix-metalloprotease MMP11[12], MMP13 [13], Kurppel-like factor KLF13 [49], c-Jun [26], ARID3B(AT-rich interactive domain 3B) [16,77], ARID3A (AT-rich interactive domain 3A) [78], germ layer specification [79] and hematopoiesis regulator LIN28A [45], growth factors such as VEGF-A [12] and IGF-II [14], and growth factor receptors like FGFR2 [80], et al.

It is necessary to mention that the same target may have different functions in different cellular processes, cellular context, and diseases. For example, ARID3B elevates the epithelial-to-mesenchymal transition (EMT) in human ovarian cancer [16], while increases cell migration in breast cancer [77]. Consequently, target genes of specific cell-type may be useful in a more targeted and precise therapy by miRNAs.

**Figure 1:**
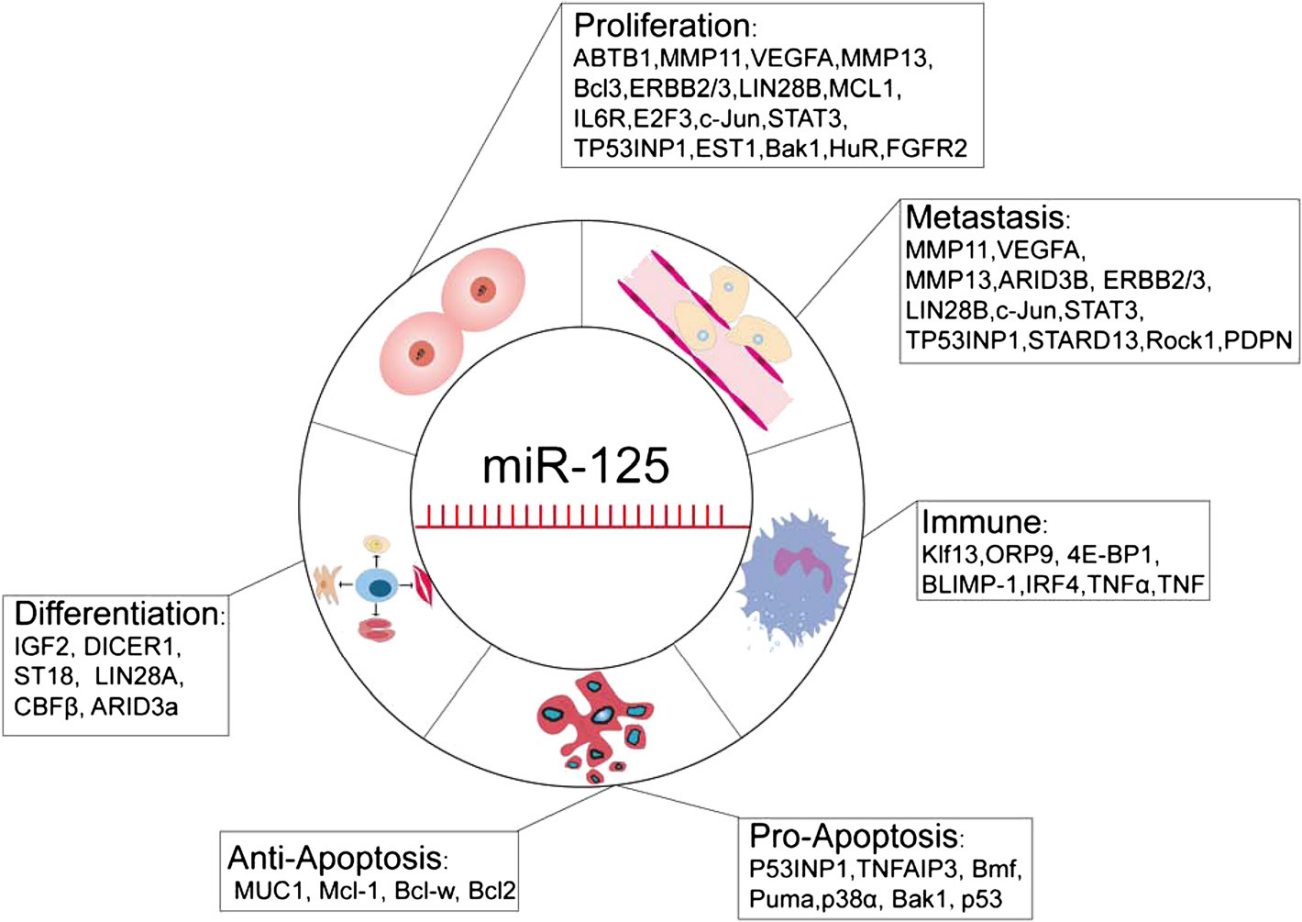
**Schematic diagram of the targets of miR**-**125 involved in different types of disease pathogenesis.**

**Figure 2:**
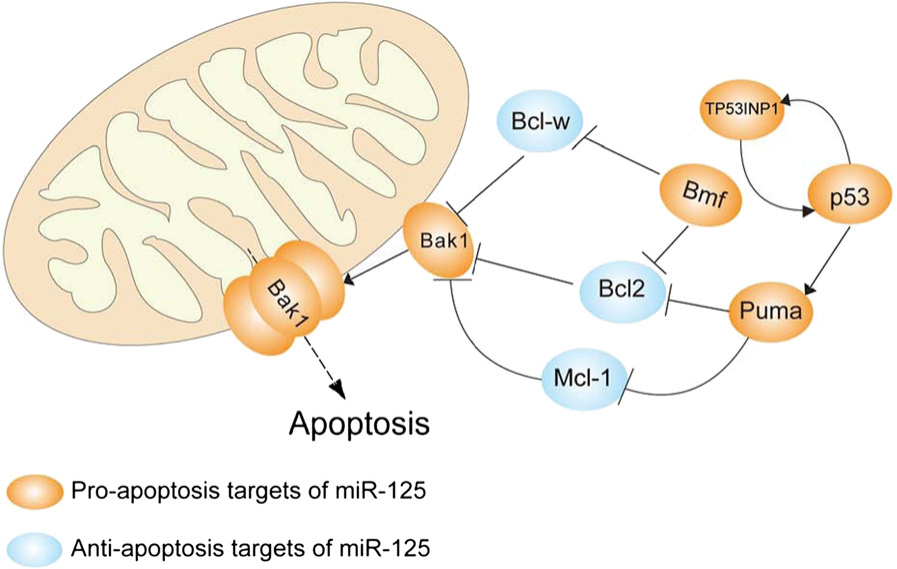
**The targets of miR**-**125 in mitochondrial apoptosis pathway.** MiR-125 plays important roles in mitochondrial apoptosis pathway by targeting pro-apoptosis or anti-apoptosis gene depends on the cell context.

As summarized in Figure 1, the targets of miR-125 family are involved in different types of diseases pathogenesis. MiR-125 can act as cancer promoter or cancer repressor depends on the cell context, among which, the mitochondrial apoptosis pathway is the well illustrated role of miR-125 with the two faces (as shown in Figure 2). The down regulated miR-125 caused breast cancer but the up regulated miR-125 induced chemoresistance, and higher expression of miR-125 promoted ALL or AML nevertheless reduced expression of miR-125b resulted in metabolic pathways transformed in CLL. Research on miR-125 in leukemia and breast cancer model may provide a new insight into current miRNA research.

### Potential of miR-125 for disease diagnosis and therapy

Some protein coding genes have been reported playing critical roles in tumor initiation and progression, and their expression levels are correlated with clinical outcome [81,82]. Growing studies demonstrate the advantages using miRNA as biomarkers for cancer and disease. The reviews above have shown that expression level of miR-125 family is distinguished in various types of disease, implying that the miR-125 could predict disease onset and might potentially serve as prognostic marker in disease-derived tissues and possibly in serum. For example, decreased miR-125a has been observed in hepatocellular carcinoma (HCC) tissues compared with matched adjacent liver tissues, and associated with patients' aggressive pathologic features [12]. Moreover, Hong and colleagues have uncovered that the expression levels of miR-125b were much lower in HCC tissues than in non-tumor liver tissues [25], indicating that both miR-125a and miR-125b have low expression level and are inversely correlated with aggressiveness and poor prognosis in HCC, and could serve as the bio-marker for HCC diagnosis and prognosis. Expression profiling of miR-125b is a potential marker for thyroid cancer as it is overexpressed in thyroid cancer and its level has remarkably distinction between benign and malignant thyroid neoplasms [83]. In human colorectal cancer, Nishida and colleagues have evaluated expression of miR-125b in 89 colorectal cancer cases, and interestingly found that the high miR-125b expression group not only showed a greater incidence of advanced tumor size and tumor invasion, but also had a significantly poorer prognosis compared to the low expression group [84], suggesting that miR-125b could be an important prognostic marker for colorectal cancer patients. The miRNA deregulation may be also associated with clinical relevance linking to key gene mutations [85]. For example, down-regulation of miR-125b was found significant correlation with TP53 mutation status [86]. Taken together, miR-125 family has great perspective as a diagnostic and prognostic biomarker. More importantly, a great amount of studies have demonstrated that circulating miRNAs in human serum and other body fluids can be used as biomarkers for cancer and other diseases [87,88]. This potential of miRNAs provides a possibility to carry out non-invasive analyses to clinicians, which can be performed easily in a large number of clinical samples. However, standard reference controls are needed in order to ensure reliable and correct measurements of miRNA levels.

Moreover, ongoing studies have also suggested the potential of the miRNA associated with therapy [89,90]. As mentioned above, miR-125 family may contribute to the initiation and progression of diseases by acting as either suppressors or promoters in a number of cancers and other diseases [16,17,22,23,25,28,29], re-introduction of synthetic miR-125 or its antisense at specific sites could become a possible treatment option.

## Conclusions

Evidences deriving from the increasing number of papers showed that members of miR-125 family play a crucial role in diverse cellular processes and many diseases especially carcinomas. It is noteworthy that the functions of miR-125 are controversial in different types of diseases. In certain contexts, miR-125 can down-regulate target oncogene as a tumor suppressor, reducing tumor proliferation and metastasis. Vice versa, a variety of studies have exhibited tumor-promoting functions of miR-125 as a tumor promoter. The advances that miR-125 and other miRNAs can be used in clinical applications exactly hold potential for future treatment of diseases. Furthermore, an increasing number of studies have identified the target genes of miR-125 family in different cellular contexts, which highlights the precise properties of this miRNA in cellular pathways and networks, especially associated with carcinoma. Moreover, miR-125 influences the fate of immune cells by targeting and regulating a set of target genes to enhance the effect against intra- or extracellular pathogens, or responding to the regulation of specific signal stimulation like IL and IFN. Since aberrant expression of miRNAs exist in almost all types of cancers and several other diseases, the potential use of miR-125 as potent prognostic markers for early diagnosis of malignancies and other diseases has drawn more and more attentions. In addition, in accordance with either disease-suppressive or - promoting properties of miRNAs in different diseases, miR-125’s ability as therapeutic agents has been delineated by many research groups. Further research is needed before miR-125 and other miRNAs can be used in clinical applications.

## Competing interests

The authors declare no competing financial interests.

## Authors’ contributions

YM S, KY L and YQ C were responsible for the conception and design of the manuscript. YM S participated in drafting the manuscript, KY L and YQ C were responsible for the review and/or revision of the manuscript. All authors read and approved the final manuscript.
